# Rapid resetting of human peripheral clocks by phototherapy during simulated night shift work

**DOI:** 10.1038/s41598-017-16429-8

**Published:** 2017-11-24

**Authors:** Marc Cuesta, Philippe Boudreau, Nicolas Cermakian, Diane B. Boivin

**Affiliations:** 1 Centre for Study and Treatment of Circadian Rhythms, Douglas Mental Health University Institute, Department of Psychiatry, McGill University, Montreal, Quebec Canada; 2 Laboratory of Molecular Chronobiology, Douglas Mental Health University Institute, Department of Psychiatry, McGill University, Montreal, Quebec Canada

## Abstract

A majority of night shift workers have their circadian rhythms misaligned to their atypical schedule. While bright light exposure at night is known to reset the human central circadian clock, the behavior of peripheral clocks under conditions of shift work is more elusive. The aim of the present study was to quantify the resetting effects of bright light exposure on both central (plasma cortisol and melatonin) and peripheral clocks markers (clock gene expression in peripheral blood mononuclear cells, PBMCs) in subjects living at night. Eighteen healthy subjects were enrolled to either a control (dim light) or a bright light group. Blood was sampled at baseline and on the 4^th^ day of simulated night shift. In response to a night-oriented schedule, the phase of *PER1* and *BMAL1* rhythms in PBMCs was delayed by ~2.5–3 h (*P* < *0.05*), while no shift was observed for the other clock genes and the central markers. Three cycles of 8-h bright light induced significant phase delays (*P* < 0.05) of ~7–9 h for central and peripheral markers, except *BMAL1* (advanced by +5h29; *P* < *0.05*). Here, we demonstrate in humans a lack of peripheral clock adaptation under a night-oriented schedule and a rapid resetting effect of nocturnal bright light exposure on peripheral clocks.

## Introduction

Shift work has been associated with a number of health consequences, such as increased risk of infection, obesity, diabetes, cardiovascular disorders, and cancer^1–5^. Atypical work schedules lead to a misalignment between the sleep-wake cycle and the endogenous circadian system, and between this system and the external environment (e.g., light-dark cycle, feeding schedule). This can lead to disturbances in various daily rhythms. Circadian disturbances are thought to be a main contributor to these shift work-associated disorders, although other factors are involved such as acute and chronic sleep deprivation, and social and familial factors. Indeed, shift work often leads to drastic changes in the sleep-wake schedule repeated over time.

Previous studies in humans showed that laboratory procedures such as simulated night shift or forced desynchrony (e.g. a 28-h day protocol) disrupt the circadian regulation of a number of physiological parameters such as leptin^6^, glucose and insulin responses after meals^6^, immune responses^7^, and cardiovascular functions^8^. Possibly, these alterations could contribute to the increased risk of medical conditions reported in shift workers such that interventions designed to alleviate circadian disturbances could have a preventive value.

In humans, the circadian system comprises a central master clock located in the suprachiasmatic nucleus (SCN) of the hypothalamus and peripheral clocks found in most tissues and cell types^9,10^. Rhythms are generated via autoregulatory feedback loops involving clock genes (e.g., *CLOCK*, *BMAL1*, *PER1*-3, and *CRY1–2*), whose expression cycles with a period of about 24 h, as observed by monitoring mRNAs levels^11^. Light being the primary synchronizer of the central circadian clock, our group previously tested its resetting potential using bright light at night both in simulated night shift conditions and in field studies with shift workers^12–15^. In simulated night shift conditions^15^, 3 cycles of 8-h bright light rapidly shifted markers of the central clock, such as plasma melatonin and cortisol. However, our preliminary studies on the expression of clock genes in a peripheral clock (peripheral blood mononuclear cells [PBMCs]) suggested that its resetting might be slower than that of the central clock^15^. Other laboratory studies in humans have addressed circadian clock gene expression after a 4-h phase advance^16^ or a 4-h delay every day for 5 days during a forced desynchrony protocol^17^. However, to our knowledge, no prior study has tested the resetting effects of bright light vs dim light exposure at night on peripheral clock gene expression, while using a simulated night shift work experiment. These are the aims of the present study.

## Results

### Plasma cortisol and melatonin rhythms and their response to a night-oriented schedule with or without bright light

**Figure 1:**
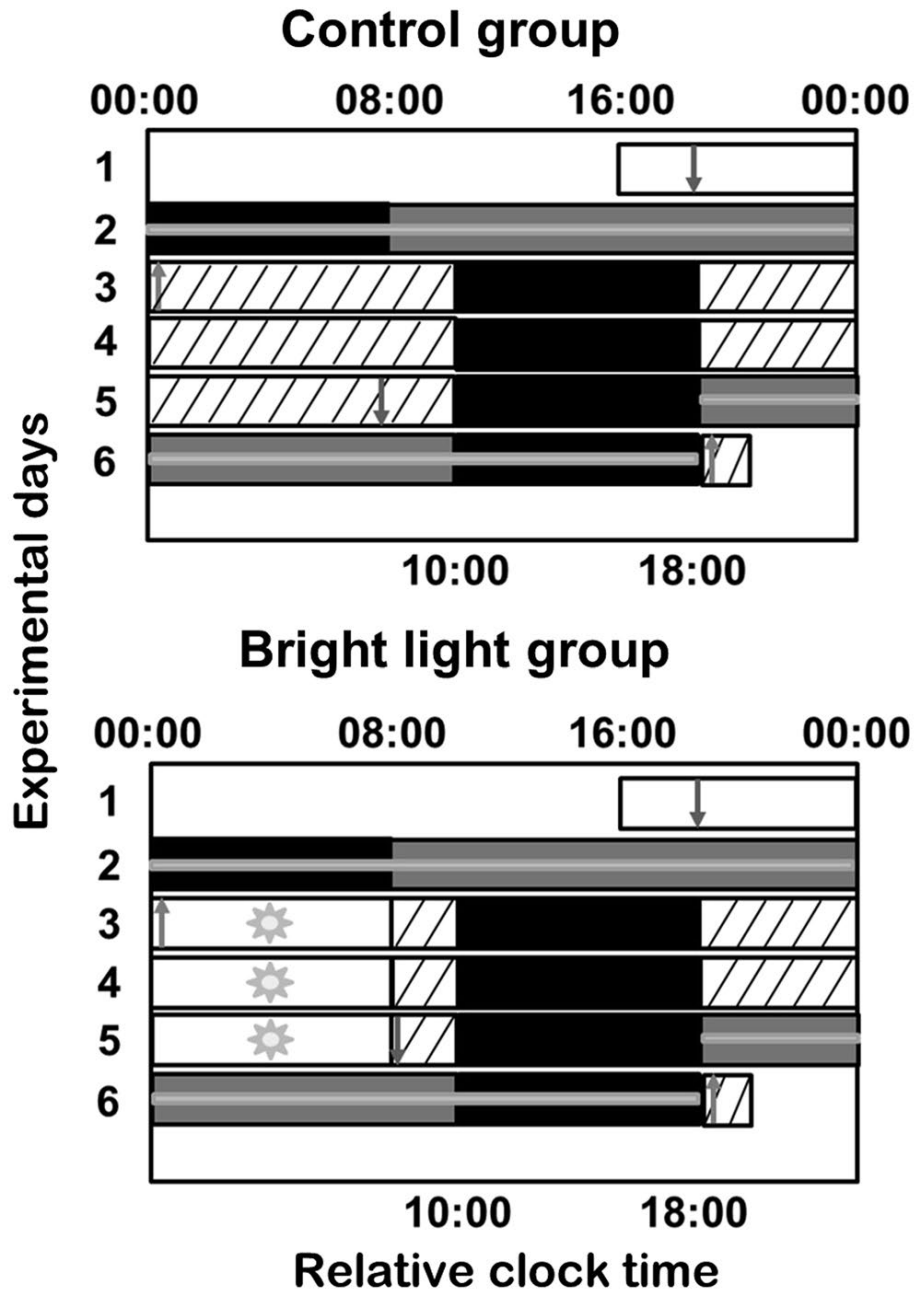
Experimental protocol. The 6-day experimental protocol is described in the Methods section. Successive experimental days are shown from top to bottom on the y-axis and time-of-day is shown on the x-axis. For analysis purposes, the bedtimes and wake times of each subject were assigned a relative clock time of 00:00 and 08:00, respectively. The black rectangles represent 8-h sleep periods in darkness. The white rectangles represent waking periods during standard days in ~100 lux. The hatched rectangles represent waking periods in dim light (~3 lux). The 8-h bright light exposure is shown as white bars with sun symbols (~6,500 lux). The grey rectangles represent the constant posture (CP) procedure (~3 lux). Blood was collected hourly for 24 h during each 16-h CP procedure and the 8-h sleep period preceding or following CP1 and CP2, respectively, as indicated by grey horizontal lines. Arrows indicate times of catheter insertion (downward arrows) and removal (upward arrows).

**Figure 2:**
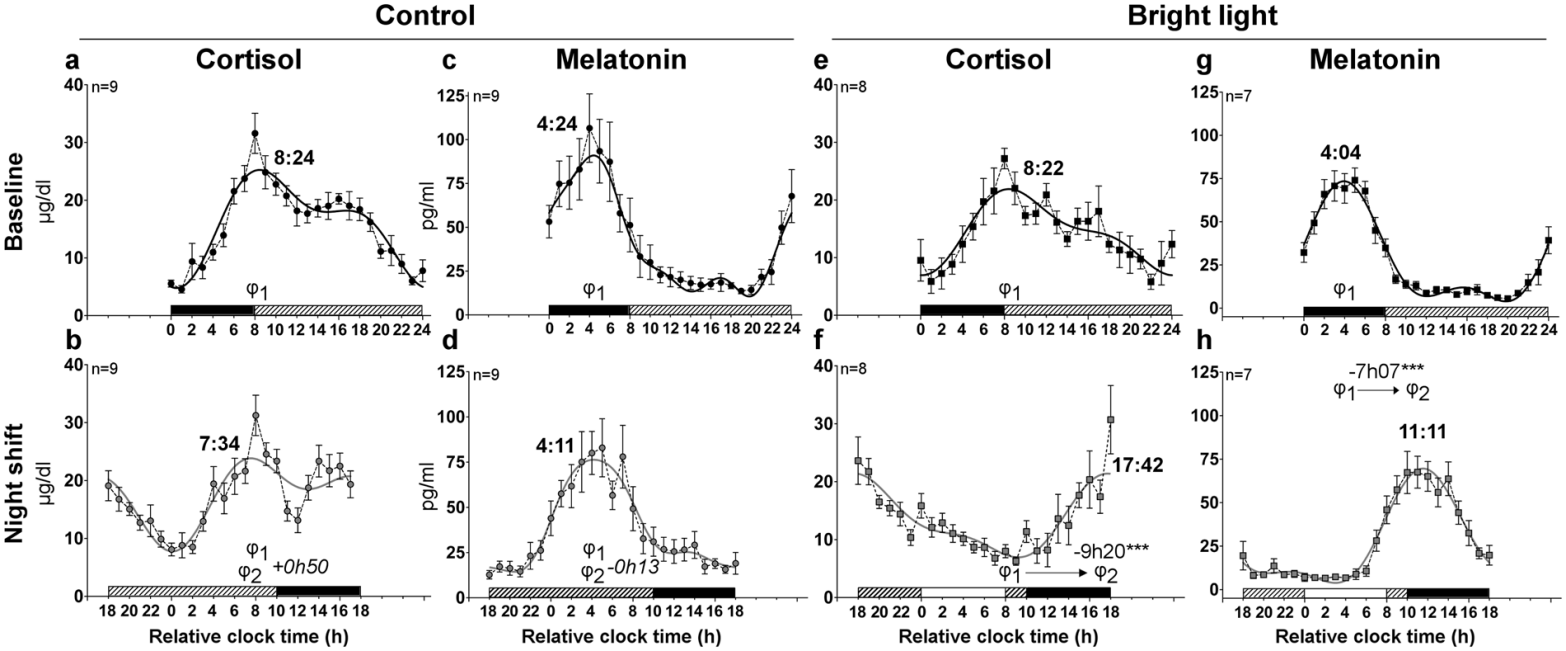
Circadian rhythms of plasma cortisol and melatonin in baseline and night shift conditions in the control and bright light groups. Mean levels ( ± S.E.M.) and group harmonic regressions of plasma cortisol (µg/dl) (**a**,**b**,**e**,**f**) and melatonin (pg/ml) (**c**,**d**,**g**,**h**). Baseline (black symbols) (**a**,**c**,**e**,**g**) and night shift (grey symbols) (**b**,**d**,**f**,**h**) conditions are represented by circles for mean levels and by lines for harmonic regressions. At baseline, the bedtimes and wake times were assigned a relative clock time of 0:00 and 8:00, respectively. Above the x-axis, the dashed, white and black bars represent the wake episodes in dim light, the bright light exposure and the sleep episode, respectively. The main phases observed at baseline and night shift conditions are represented by φ1 and φ2, respectively, and are indicated as hours:minutes over the curves. The position of both phases is illustrated (**b**,**d**,**f**,**h**) allowing a visualization of their difference, along with the value and the statistical significance of the phase shift. ***P < 0.001.

At baseline, under a day-oriented schedule (Fig. 1), central markers plasma cortisol and melatonin were rhythmic in all individuals and at the level of the groups (Fig. 2a,c,e,g; Table 1). For melatonin and cortisol rhythms, baseline phases and amplitudes were similar between control and bright light groups (*P* ≥ 0.93) and the phases occurred at conventional times-of-day: the melatonin levels peaked during the nocturnal sleep episode, whereas the peak of cortisol occurred shortly after lights on.

**Table 1:** Circadian phase of central and peripheral markers.

Marker	Treatment	Condition	Phase ± SEM (h:min) (nonlinear mixed procedure)	Individual regressions (n significant/n total)
Cortisol	Control	Baseline	8:24 ± 0:13	9/9 (100%)
		Night shift	7:34 ± 0:21	9/9 (100%)
	Bright light	Baseline	8:22 ± 0:23	8/8 (100%)
		Night shift	17:42 ± 0:18	8/8 (100%)
Melatonin	Control	Baseline	4:24 ± 0:40	9/9 (100%)
		Night shift	4:11 ± 1:12	9/9 (100%)
	Bright light	Baseline	4:04 ± 0:37	7/7 (100%)
		Night shift	11:11 ± 0:33	7/7 (100%)
*PER1*	Control	Baseline	7:49 ± 0:13	8/9 (89%)
		Night shift	11:00 ± 0:43	4/9 (44%)
	Bright light	Baseline	7:46 ± 1:02	4/9 (44%)
		Night shift	15:09 ± 1:00	7/8 (87.5%)
*PER2*	Control	Baseline	8:44 ± 0:39	4/9 (44%)
		Night shift	9:45 ± 1:41	2/9 (22%)
	Bright light	Baseline	6:06 ± 0:34	6/9 (67%)
		Night shift	15:30 ± 0:42	3/8 (37.5%)
*PER3*	Control	Baseline	6:10 ± 0:18	8/9 (89%)
		Night shift	5:33 ± 0:35	6/9 (67%)
	Bright light	Baseline	5:22 ± 1:10	8/9 (89%)
		Night shift	14:16 ± 1:02	7/8 (87.5%)
*BMAL1*	Control	Baseline	15:11 ± 0:31	8/9 (89%)
		Night shift	17:38 ± 0:52	5/9 (56%)
	Bright light	Baseline	21:03 ± 0:55	5/9 (56%)
		Night shift	15:34 ± 1:44	3/8 (37.5%)
*REV-ERBα*	Control	Baseline	2:08 ± 0:20	9/9 (100%)
		Night shift	2:32 ± 0:36	7/9 (78%)
	Bright light	Baseline	3:27 ± 0:33	6/9 (67%)
		Night shift	9:49 ± 1:09	6/8 (75%)

Under the night-oriented schedule, cortisol and melatonin group profiles remained significantly rhythmic in both groups (Fig. 2b,d,f,h; Table 1). The shape of the rhythms was similar to baseline, except for cortisol in the control group (Fig. 2b). Indeed, in the final assessment, we observed two peaks for plasma cortisol. The first peak occurred at 7:34 ± 0:21 (reported in Table 1) and was followed by a rapid decrease in cortisol levels shortly after lights off. Levels remained low until 12:00, and increased again to reach a second peak of smaller amplitude at 16:53 ± 0:37.

The phases of plasma cortisol (the first peak) and melatonin were not significantly shifted in the control group (Fig. 2a–d; *P* = 0.06 and *P* = 0.86, respectively), while in response to bright light, both cortisol and melatonin rhythms were significantly delayed (−9h20 and −7h07, respectively *P* < 0.001 for both; Fig. 2e–h). In both groups, the amplitudes of central markers were not modified under a night-oriented schedule (*P* ≥ 0.12; Table 2), except for the group cortisol rhythm amplitude that was reduced in the control group (*P* = 0.04; Table 2).

**Table 2:** Circadian amplitude of central and peripheral markers.

Marker	Treatment	Condition	Amplitude (nonlinear mixed procedure)	*P* value
Cortisol	Control	Baseline	7.7 ± 0.6	0.04
		Night shift	5.6 ± 0.6	
	Bright light	Baseline	6.8 ± 0.7	0.54
		Night shift	6.2 ± 0.7	
Melatonin	Control	Baseline	35.4 ± 2.7	0.12
		Night shift	28.9 ± 2.7	
	Bright light	Baseline	28.2 ± 1.7	0.89
		Night shift	27.9 ± 1.7	
*PER1*	Control	Baseline	0.9 ± 0.1	0.06
		Night shift	0.5 ± 0.1	
	Bright light	Baseline	0.4 ± 0.1	0.85
		Night shift	0.5 ± 0.1	
*PER2*	Control	Baseline	0.7 ± 0.1	0.03
		Night shift	0.3 ± 0.1	
	Bright light	Baseline	0.8 ± 0.1	0.46
		Night shift	0.6 ± 0.1	
*PER3*	Control	Baseline	1.2 ± 0.1	0.004
		Night shift	0.6 ± 0.1	
	Bright light	Baseline	0.4 ± 0.1	0.52
		Night shift	0.5 ± 0.1	
*BMAL1*	Control	Baseline	0.8 ± 0.1	0.07
		Night shift	0.5 ± 0.1	
	Bright light	Baseline	0.5 ± 0.1	0.23
		Night shift	0.3 ± 0.1	
*REV-ERBα*	Control	Baseline	1.1 ± 0.1	0.003
		Night shift	0.6 ± 0.1	
	Bright light	Baseline	0.8 ± 0.1	0.04
		Night shift	0.4 ± 0.1	

### Rhythms of clock gene expression in PBMCs and their response to a night-oriented schedule with or without bright light

**Figure 3:**
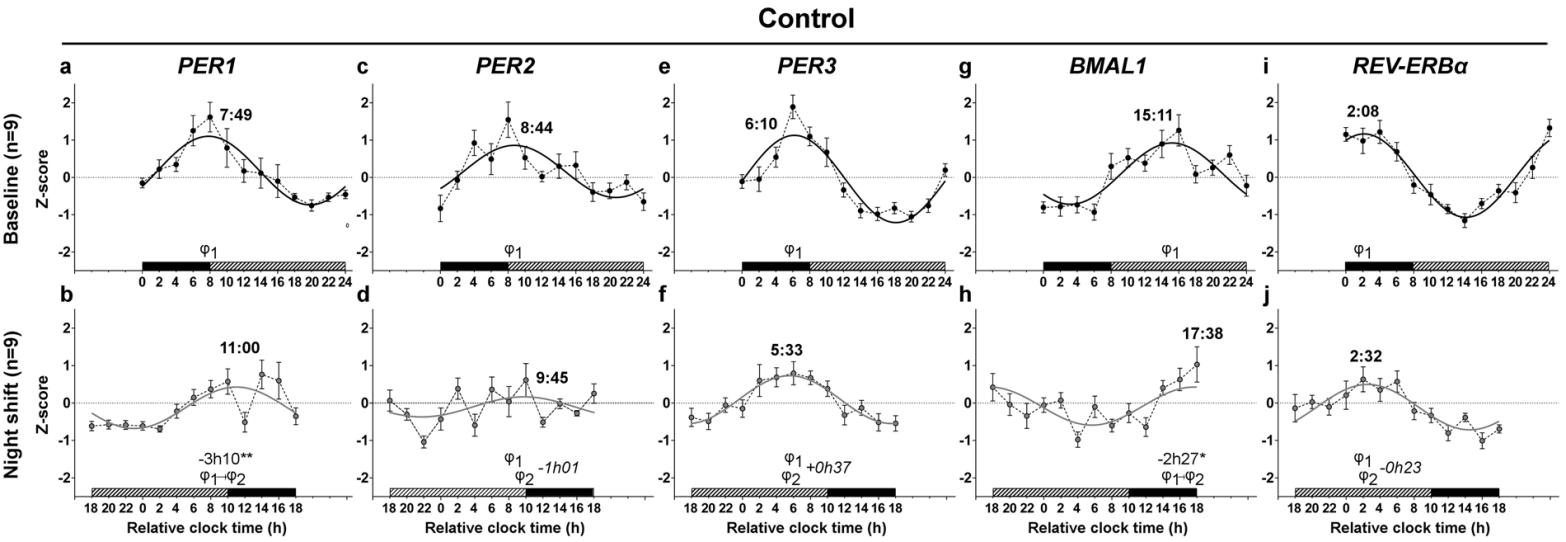
Circadian rhythms of clock gene mRNA expression in PBMCs in baseline and night shift conditions in the control group. Mean levels (±S.E.M.) and group harmonic regressions of *PER1* (**a**,**b**), *PER2* (**c**,**d**), *PER3* (**e**,**f**), *BMAL1* (**g**,**h**) and *REV-ERBα* (**i**,**j**) are given as Z-scores. Results are presented for the baseline (**a**,**c**,**e**,**g**,**i**) and night shift (**b**,**d**,**f**,**h**,**j**) conditions. See the legend of Fig. 2 for details. *P < 0.05; **P < 0.01.

**Figure 4:**
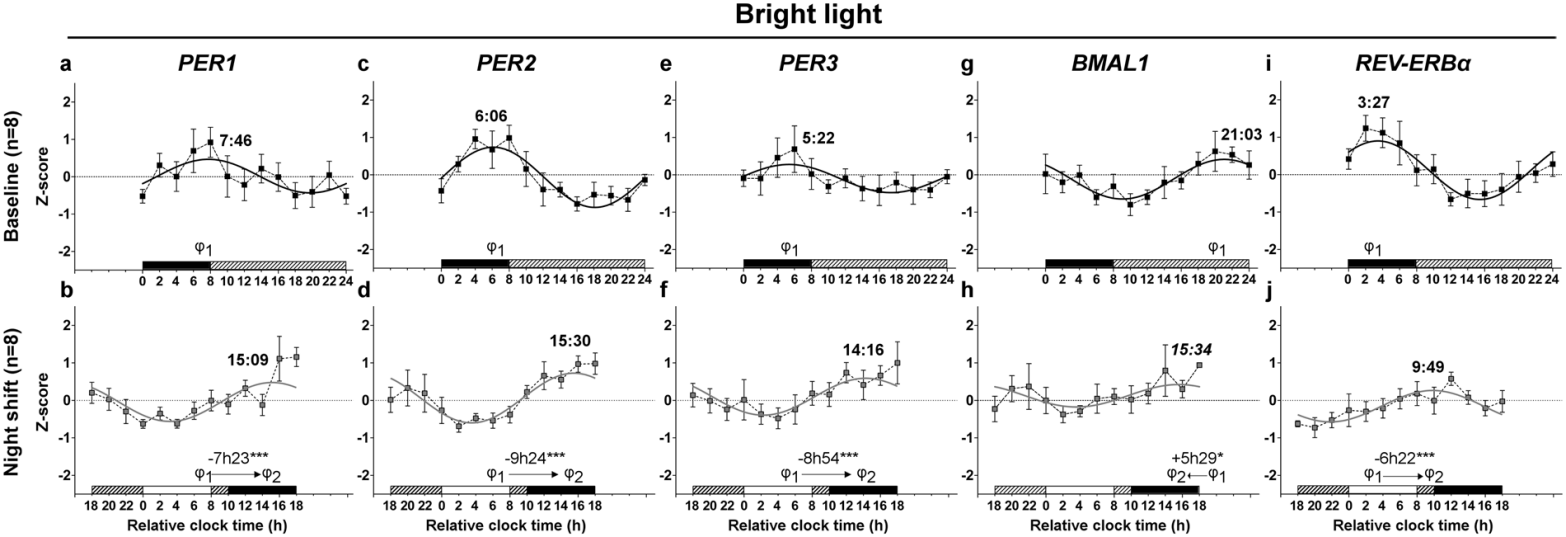
Circadian rhythms of clock gene mRNA expression in PBMCs in baseline and night shift conditions in the bright light group. Mean levels ( ± S.E.M.) and group harmonic regressions of *PER1* (**a**,**b**), *PER2* (**c**,**d**), *PER3* (**e**,**f**), *BMAL1* (**g**,**h**) and *REV-ERBα* (**i**,**j**) are given as Z-scores. Results are presented for the baseline (**a**,**c**,**e**,**g**,**i**) and night shift (**b**,**d**,**f**,**h**,**j**) conditions See the legend of Fig. 2 for details. *P < 0.05; ***P < 0.001.

At baseline, group regressions indicated the presence of significant circadian rhythms of RNA expression for the clock genes *PER1*, *PER2, PER*3, *BMAL1* and *REV-ERBα* in the control and bright light groups (*P* < 0.01 for all; Fig. 3a,c,e,g,i and 4a,c,e,g,i; Table 1). In addition, the percentage of individuals expressing significant rhythms ranged between 44% and 100% (Table 1) and percentages were similar between the two groups for all clock genes (*P* ≥ 0.08), except for a trend (*P* = 0.06) with more individuals expressing significant *PER1* rhythms in the control (8 out of 9) vs. the bright light group (4 out of 9). For both group (Figs 3 and 4) and individual rhythms (Figs S1 and S2), the acrophases occurred at conventional times-of-day (at the beginning of the nocturnal sleep episode for *REV-ERBα;* around lights on for *PER1-*3; in the middle to late afternoon for *BMAL1*).

Between-group analyses revealed there was no phase difference for *PER1, PER3* and *REV-ERBα* between the control and bright light groups at baseline (*P* ≥ 0.07), while the *PER2* and *BMAL1* rhythms peaked earlier and later in the bright light group, respectively (*P* < 0.01). The amplitude of *PER2* and *BMAL1* rhythms was similar in both groups (*P* ≥ 0.11) while it was higher in the control group for *PER1* (*P* < 0.05), *PER3* (*P* < 0.001) and *REV-ERBα* (*P* < 0.05) rhythms.

Under the night-oriented schedule, all rhythms were significant at the level of the group (*P* < 0.05 for all; Figs 3b,d,f,h,j and 4b,d,f,h,j; Table 1) The percentage of individuals expressing significant rhythms was higher for *PER1*, *PER3* and *REV-ERBα* (ranging from 44% to 87.5%) than for *PER2* (22% and 37.5% in the control and bright light groups, respectively) and *BMAL1* (56% and 37.5% in the control and bright light groups, respectively) (Figs S1 and S2; Table 1). In both the control and bright light groups, the percentage of individuals expressing significant clock gene rhythms did not decrease significantly (Table 1; *P* ≥ 0.13) following night shifts, except for a trend in *PER1* (Table 1; *P* = 0.06). In addition, percentages were similar between the two groups for all clock genes following night shifts (Table 1; *P* ≥ 0.13). The shape of the rhythms was similar to baseline, except for *PER1* in the control group (Fig. 3b), which displayed a pattern similar to that of cortisol. Therefore, we performed a cross-correlation analysis between the pattern of cortisol and that of *PER1* revealing that the best correlation was found when both cortisol and *PER1* rhythms were in phase with each other. In other words, cortisol levels and *PER1* expression increased and decreased at the same time.

Following night shifts in the control group, the phase of *PER1* and *BMAL1* rhythms was delayed by −3h10 (*P* < 0.01) and −2h27 (*P* < 0.05), respectively (Fig. 3b,h), while no significant shift was observed for the other clock genes (*P* ≥ 0.38). In contrast, in response to bright light exposure at night, the expression of all clock genes was significantly delayed by ~7–9 h (*PER1*: −7h23; *PER2*: −9h24; *PER3*: −8h54; *REV-ERBα*: −6h22; *P* < 0.001 for all), except that of *BMAL1*, which was significantly advanced by +5h29 (*P* < 0.05). This was confirmed by the comparison of the phases observed in the control and bright light groups under the night-oriented schedule. All the clock gene peaked significantly later in the bright light group than in the control group (*P* ≤ 0.01), except *BMAL1*, for which no between-group differences were detected (*P* = 0.29). As illustrated in Fig. S2, a visual analysis of individual regressions suggested the presence of a substantial interindividual variability for the resetting of the *BMAL1* rhythm. Indeed, while the *BMAL1* rhythm of some individuals displayed phase advances in response to bright light (S01, S02, S05), phase delays (S06, S13) or no shift (S04, S10, S11) were observed in other individuals. Some level of interindividual variability was also observed for *PER2* rhythm, mainly due to the lack of significant rhythms following night shifts in several individuals (Fig. S2).

In the control group, under a night-oriented schedule, the amplitude of *PER2* (*P* < 0.05), *PER3* (*P* < 0.01) and *REV-ERBα* (*P* < 0.01) group rhythms was significantly reduced (Table 2), while the amplitude of all clock gene rhythms was similar to baseline (*P* ≥ 0.22; Table 2) in the bright light group, except for *REV-ERBα* which was dampened (*P* < 0.05; Table 2). In addition, the between-group analysis revealed no amplitude difference (*P* ≥ 0.25), except for *PER2* rhythm displaying a higher amplitude in the bright light group at the end of study (*P* = 0.05).

## Discussion

In the present simulated night shift study, we showed a rapid resetting by nocturnal bright light exposure of both central and peripheral circadian clocks following three cycles of 8-h bright light exposure at night. This was in contrast with the lack of adjustment of these clocks under night shift in the control group. To our knowledge, this is the first demonstration of a rapid resetting effect of light on peripheral clock genes in humans.

Circadian rhythms of central and peripheral clock markers were observed at baseline with phases similar to those previously reported^15,17–24^. The phase of each clock gene was similar between individuals in both groups, except for *PER2* and *BMAL1*, which occurred earlier and later, respectively in the bright light group. The difference was moderate for *PER2* (2h38) and in the range of what has been reported previously^21^ using the same type of methodology and analysis. The difference was more pronounced for *BMAL1* (5h53) which is not surprizing considering the high variability reported for this clock gene in previous studies^15,17–24^. This variability was confirmed in the present study, with *BMAL1* expression occurring in the middle and late afternoon in the control and bright light groups, respectively.

We showed that an abrupt 10-h delay shift in the timing of sleep results in a decreased amplitude of clock gene rhythms, sometimes leading to the loss of detectable rhythms as it was reported under different sleep disruption paradigms^17,20,24^. In addition, we demonstrated in the control group that night shift induces small phase shifts of *PER1* and *BMAL1* rhythms, while *PER2, PER3* and *REV-ERBα* were not shifted. This led to a loss of temporal coordination between the different clock components within PBMCs peripheral clocks. The phase and amplitude changes induced by the night-oriented schedule could be due to acute sleep deprivation, changes in the timing of the sleep-wake and in the feeding patterns, or a combination of these elements. For instance, as we mentioned earlier^20^, sleep deprivation can induce a reduction of the amplitude of circadian rhythms. This can lead in some cases to a complete loss of rhythm indicating an involvement of this factor alone or in combination with circadian misalignment.

Both plasma melatonin and cortisol were rhythmic and did not adjust to a night-oriented schedule in the control group. We could have expected these rhythms to free run in dim light conditions. However, as only 3 days separated the initial and final assessments, phase delays due to free run would be small, and did not lead to a significant difference between assessments. Interestingly, in the control group, the shape of the cortisol rhythm was altered following simulated night shifts. The acrophase of plasma cortisol rhythm normally seen soon after lights on under a day-oriented schedule (i.e. 8:00 in our protocol) was still present under a night-oriented schedule (and therefore not shifted). However, it was followed by a rapid decrease in cortisol levels shortly after lights off in the morning (i.e. 10:00 in the night shift part of our protocol). This drop in cortisol levels lasted for a few hours before the levels rose again to reach a second peak around 17:00, close to the shifted time of awakening (lights on occurred at 18:00 under the night-oriented schedule). This bimodal pattern of cortisol secretion could be the result of the displacement of the sleep episode into the daytime in the absence of a concomitant shift of the endogenous circadian pacemaker. Indeed, it was hypothesized that cortisol release is inhibited by sleep, although contradictory results exist (reviewed in^25^). This bimodal pattern of cortisol could also be interpreted as the result of a dissociation of central and peripheral mechanisms known to regulate the cortisol rhythm^21,26^. The first cortisol peak of high amplitude observed shortly after lights off of the daytime sleep period could be controlled by the central clock as its phase is similar to that observed under a day-oriented schedule. The second cortisol peak of moderate amplitude observed around 17:00 could be driven by peripheral mechanisms at waketime including an involvement of the adrenal clock. Indeed, in mice, adrenal glucocorticoids have a role in the resynchronization after a shift in the light-dark cycle^27^, and retinohypothalamic signals can be relayed to the adrenal clock via a pathway bypassing the SCN^28–30^. If such mechanisms exist in humans, they could underlie effects of night shift on cortisol and peripheral clock rhythms.

Interestingly the pattern of *PER1* expression in PBMCs was correlated to that of plasma cortisol under a night-oriented schedule. We and others demonstrated previously that a single administration of exogenous cortisol in humans is associated with an acute increase of *PER1* expression in PBMCs^21,31^. The present study revealed that a decrease in plasma cortisol is associated with a decrease of *PER1* expression which reinforces the hypothesis that *PER1* acts as an early-response gene to glucocorticoids^21^.

Here, we confirmed previous studies using bright light at night in both real and simulated night shift conditions^12–15^ by showing that plasma cortisol and melatonin rhythms were almost fully reset to a night-oriented schedule by 3 consecutive cycles of 8-h bright light exposure at night (~7–9 h shift). The rhythms of most clock genes examined were also reset to a similar degree. In our previous study^15^, we showed the alignment of *PER1/2* gene expression to a night-oriented schedule only after 10 cycles of 8-h bright light exposure rather than 3 cycles as reported here. A higher degree of inter-individual variability and the lack of significant rhythms in earlier parts of the experiment had prevented us from detecting more rapid shifts. The difference between our prior study and the present work is likely to be the result of a refinement of our technique of circadian clock gene analysis, the addition of subjects in the bright light group, and the use of a mathematical model (nonlinear mixed-effect procedure) allowing an analysis at the level of the group while taking into account the repeated nature of our experimental design in each individual.

The present experimental paradigm did not allow us to clarify which part of the circadian system regulated the resetting of the core clock components in PBMCs. It is likely that their resetting was controlled by the SCN as the magnitude of the shifts were similar between central and peripheral markers. However, it is also possible that light affected the peripheral clocks via a retino-hypothalamic pathway bypassing the SCN, as recently proposed^28,29,32^. In the bright light group, the phase of the *BMAL1* group rhythm was advanced by ~5 h, which is different than the resetting observed for the other clock genes in response to bright light. This difference can be explained by the individual variability in *BMAL1* rhythms. Indeed, the *BMAL1* rhythm of some subjects was phase advanced in response to bright light, while phase delays or no shift were observed in other subjects as seen in Fig. S2. Different pace of resetting among core clock components has already been described for instance in the mouse SCN in response to an advance in the lighting schedule^33^ and could be seen as a rate-limiting factor in adjustment to abrupt changes of the light-dark and sleep-wake cycles. While we observed a dissociation of mRNA expression of clock genes, the functional implication of such a finding is unclear as the clock feedback loop could still be functional even with a dampened rhythm of *BMAL1* gene expression. Indeed, it was shown that constitutive *Bmal1* expression is able to rescue circadian behavioral rhythms in *Bmal1* ^−/−^ mice^34^. In addition, *Rev-erbα* KO mice, which have a low amplitude of *Bmal1* mRNA rhythm, show robust circadian rhythms^35^. We cannot totally exclude the possibility that baseline differences in the timing of *BMAL1* rhythm could have contributed to the between group differences in the phase shift of its rhythm. That said, in response to bright light, a phase delay and advance of *PER2* and *BMAL1* were observed following night shifts, respectively. Therefore, it is unlikely that differences at baseline contributed to between-group differences in phase shift. Moreover, the final phase of *PER1*-*3* and *REV-ERBα* genes occurred significantly later in the bright light group, confirming our interpretation.

The present study has a few limitations. We only described peripheral resetting for one set of peripheral clocks (i.e. PBMCs) and further studies are required to evaluate whether other peripheral clocks can also be quickly reset by bright light exposure. Our study was performed in simulated night-shift conditions in which we controlled a series of parameters such as levels of light exposure, food intake, activity levels, and the timing of sleep. Studies in real night-shift conditions are needed to confirm that peripheral circadian markers can be quickly reset by exposure to phototherapy in field conditions and their degree of resetting across various tissues. To our knowledge, only a few studies so far have looked at the expression of clock genes under night shift conditions either using two time points^36^, or studying only one clock gene over 24 h^37^. Both studies showed alteration of clock gene expression and these results need to be confirmed by more extensive studies. In addition, rhythmicity extends beyond circadian clock genes and future work should study a larger group of genes using a genome wide approach. Such an approach would provide additional information on the participation of genetic disturbances to the adverse health outcomes of atypical to work schedules.

In conclusion, we demonstrated that on a night-oriented schedule, molecular components of a human peripheral clock can be rapidly reset by bright light exposure with direction and magnitude comparable to those of markers strongly controlled by the central clock. This challenges the hypothesis that peripheral clocks are slower to adjust to a new schedule compared to the central clock, although more peripheral tissues and earlier time points should be tested to confirm this. The present study suggests that phototherapy could be considered as a non-pharmacological intervention to counteract the deleterious effects of shift work or jet lag as it allows a more complete and rapid adjustment of different parts of the circadian system. Nevertheless, more studies on the effects of bright light are needed to evaluate the balance between positive and negative effects on health. Indeed, shift work has been associated with an increase incidence of breast cancer^38^. As disruption of circadian rhythms is believed to be linked with this increase risk, properly timed bright light exposure could be pursued as a preventive approach through its action on the circadian system. However, it was suggested that an excess of bright indoor light at night could also lead to an increase in breast cancer risk through its inhibitory effect on melatonin secretion at night^39^. That said, there is a keen interest to develop new treatments to alleviate the misalignment of the circadian system observed in shift workers. Indeed, amplitude and phase alterations of the core clock components as seen in the present study might be involved or associated with the negative effects of atypical sleep-wake schedules on health. Although circadian misalignment is not the only contributing factor to shift work maladaptation, it remains possible that correcting for it might reduces the risk of developing several of these medical complications.

## Methods

### Subjects

A 6-day within-subject simulated night shift procedure was conducted in time isolation at the Centre for Study and Treatment of Circadian Rhythms of the Douglas Mental Health University Institute, McGill University. A total of 19 young healthy and drug-free participants (23.7 ± 4.2 years old; 2 women) were enrolled to the study. Eighteen of 19 subjects participated in the full 6-day experiment (S20 only participated to the baseline part of the study and thus, his results were excluded from all analyses). They were recruited as previously described^18^. Study procedures and written consent forms were approved by the Douglas Institute Ethic Board and the study was within the ethical standard of the declaration of Helsinki. A written informed consent was obtained from all participants. Each enrolled subject participated to either a control (n = 10, 1 woman) or bright light group (n = 9, 1 woman). Most of the subjects (n = 14) participated to the study from May to August in 2012, 2013 and 2014 (Table S1). Data obtained from this study for plasma melatonin have been published previously^7,40^. In addition, we used plasma melatonin and cortisol data, as well as PBMC samples obtained from 5 subjects during a 12-day study in 2005 (Table S1)^15^. The 6 first days of this study protocol were the same than those from the 2012–2014 study described here. Thus, subjects were assigned to study groups in a non-randomized manner. During the experiment, blood samples from two subjects from the bright light group (S12 for all parameters following night shifts and S06 for plasma melatonin at baseline) could not be obtained at certain time points due to difficult draws or other technical issues preventing statistical assessment of rhythmicity. Thus, we used data from 17 subjects for analyses of plasma cortisol and clock gene expression (n = 9 for the control group and n = 8 for the bright light group) and from 16 subjects for analyses of plasma melatonin (n = 9 for the control group and n = 7 for the bright light group). Age (23.0 ± 3.4 and 25.3 ± 4.6; *P* = 0.41), BMI (21.3 ± 1.2 and 22.5 ± 1.6; *P* = 0.10) and chronotype (53 ± 2 and 56 ± 7; *P* = 0.64) of the remaining 17 participants were similar between the control and bright light groups, respectively (Table S1). The two women were naturally ovulating as confirmed by measuring progesterone levels around day 21 of their menstrual cycle. They had regular menses of 32 ± 1 day and 27 ± 1 day and entered the laboratory on the 2^nd^ and 5^th^ day, respectively, after the start of menses. They were thus studied during the follicular phase of their menstrual cycle.

### Experimental protocol

The experimental protocol was adjusted to each participant’s habitual sleep-wake cycle. Before laboratory admission, subjects maintained a stable 8-h nocturnal sleep schedule for at least 7 days in order to stabilize their circadian system to their schedule^18^. The average sleep duration was similar (*P* = 0.15) in the control (07h59 ± 00h02) and bright light groups (08h03 ± 00h02). The sleep-wake schedule in laboratory was calculated using sleep diaries filled out by the subjects and by their calls made to the laboratory at each bed and wake time. In addition, actigraphic recordings (Actiwatch-L, Mini-Mitter, Bender, OR, USA) served as an objective sleep measure.

Subjects arrived at the time-free laboratory and spent their 1^st^ experimental evening and nocturnal 8-h sleep episode according to their habitual sleep/wake schedule (Fig. 1)^15^. This was followed by a 16-h wake episode in constant posture (CP) conditions in which levels of activity, light exposure and food intake were controlled to minimize their masking on observed circadian rhythms^18^. The first sampling session served to determine baseline rhythms and was done for 24 h under a day-oriented schedule during the 8-h nocturnal sleep episode followed by a first 16-h CP procedure. Experimental day 3 started with a night of sleep deprivation in which participants were allowed to move. This was the 1^st^ simulated night shift cycle. It was followed by an 8-h daytime sleep episode that was delayed by 10 h relative to the habitual sleep time. This night-oriented schedule was maintained for 4 consecutive 24-h cycles until the end of the study (experimental days 3 to 6). A second 16-h CP procedure (experimental days 5 and 6) took place in simulated night shift conditions, starting after the third diurnal 8-h sleep episode of experimental day 5. This 16-h CP procedure followed by the fourth diurnal 8-h sleep episode represented the second 24-h sampling session. It was used to determine the effects of a night-oriented schedule in both study groups. Participants left the laboratory upon awakening from the last diurnal sleep episode on experimental day 6.

On experimental day 1, subjects were given an evening meal and a snack 2 h before lights off. During experimental day 2 (first CP) and the first night shift (experimental day 3), subjects received hourly isocaloric snacks. From waketime on experimental day 3 to bedtime on experimental day 5, three meals per day were provided (45 min, 4 h, and 10 h after lights on), as well as a snack 2 h before lights off. From waketime on experimental day 5 to bedtime on experimental day 6, (second CP), subjects were given hourly isocaloric snacks.

### Light intensity and exposure

During the 6-day experiment, light intensity was tightly controlled and measured every 2 h in the participants’ average angle of gaze during waking periods with a research photometer (IL1400A, International Light Technologies Inc., Peabody, MA, USA). During all sleep episodes, lights were turned off and intensity was below 0.1 W/m^2^ (0.03 lux) as verified via the light-mounted sensor of the wrist actigraphic device (Actiwatch-L, Mini-Mitter, Bender, OR, USA). Upon arrival on experimental day 1, subjects remained under ordinary indoor room light with average ambient light levels similar (*P* = 0.51) in the control (295.5 ± 10.8 W/m^2^ or 100.9 ± 3.7 lux) and the bright light group (329.2 ± 52.4 W/m^2^ or 112.4 ± 17.9 lux). From the start of CP1 to the end of the study, all wake periods were in very dim light and similar in both groups (control: 7.6 ± 0.6 W/m^2^ or 2.6 ± 0.2 lux; bright light: 10.5 ± 1.2 W/m^2^ or 3.5 ± 0.4 lux; *P* = 0.06), except during the intervention for the bright light group.

During the 8-h bright light exposure (starting 10 h before bedtime), subjects were seated and asked to center their gaze on an image hung on the wall for 10 min every 20 min (considered as the “up” period)^15^. During this “up” period, subjects of the bright light group were exposed to an average of 18,742 ± 586 W/m^2^ (6,422 ± 185 lux). The angle of gaze was not restricted the rest of the time (“down” period) and the light intensity measured at that time represented about 80% of the averaged intensity observed during “up” periods. In the control group, the same angle of gaze procedure was used, but subjects were kept in dim light (7.8 ± 0.6 W/m^2^ or 2.7 ± 0.2 lux).

### Sampling and processing of central markers

An indwelling catheter was inserted in a forearm vein of each participant at least 4 h prior to start of each CP procedure (in the evening of experimental day 1 for CP1 and the morning of experimental day 5 for CP2)^18,21,41^. This procedure was followed in order to minimize masking effects due to the stress induced by the catheter insertion. This system allowed blood sampling for extended periods without disturbing the subject’s sleep. Blood sampling started 15 min after the beginning of CP1 and CP2 and ended after 24 h. Every ~60 min, 2 ml of whole blood was withdrawn and transferred in K_2_EDTA-coated tubes. Plasma samples were obtained after centrifugation at 1,494 g for 15 min at 4 °C and stored at −80 °C until further analysis.

Plasma melatonin levels were quantified in duplicates using radioimmunoassay and following manufacturer’s instructions. The kit used in the 2012–2014 study (Labor Diagnostika Nord, Nordhorn, Germany; range 3–1000 pg/mL; sensitivity, 2.3 pg/mL) differed from the kit used in 2005 (Stockgrand, Guilford, Surrey, UK; range 5–500 pg/mL; sensitivity 2.5 pg/mL). The intra- and inter-assay coefficients of variability (CVs) were of 8.3% and 4.4%, respectively. Since the circadian phase was our main parameter and to verify that the possible variation introduced by the use of different kits did not impact our data, we transformed our data into Z-score (Z = (x − μ)/σ where x is the raw value, μ is the mean and σ is the standard deviation). We found the same results and statistical differences than those observed using raw data such that we decided to report the raw data results.

Plasma cortisol levels were also quantified in duplicates using radioimmunoassay (MP Biomedicals, Montreal, QC; range 1.0–100 μg/dL; intra- and inter-assay CVs were of 7.9% and 4.1%, respectively).

### Sampling and processing of peripheral markers

A total of 10 ml of whole blood was collected in two heparin-coated tubes (5 ml in each tube) every ~120 min, and centrifuged for 30 min at 1600 rpm (370 g) on a density gradient (Histopaque-1077, Sigma Aldrich, Oakville, ON, Canada). Isolated PBMCs were washed 3 times with 1X phosphate-buffered saline (PBS), lysed in Trizol (Life Technologies, Burlington, ON, Canada) and stored at −80 °C until processing.

RNA was extracted from PBMCs samples from the 2012–2014 study and leftover samples from the 2005 study (other samples were used for clock gene expression analysis by real-time PCR in a previous study from our laboratory^15^). Once total RNA was extracted, we verified its concentration and purity using a NanoDrop 1000 spectrophotometer (Thermo Fisher, Ottawa, ON, Canada). When we detected impurities, we decontaminated the samples with RNeasy Mini Kit (Qiagen, Toronto, ON, Canada). We also assessed integrity of total RNA and contamination with genomic DNA on agarose gels. In case of DNA contamination, we treated the samples with RNase-Free DNase (Qiagen) followed by another RNA purification (RNeasy Mini Kit).

Total RNA was set at 20 ng/μl and reverse transcribed with High Capacity cDNA Reverse Transcription Kit (Life Technologies). The obtained cDNA was used in real-time PCR using TaqMan Gene Expression Assays (Life Technologies) on an AB7500 Real-Time PCR System (Life Technologies) and data were analyzed with the 2^−ΔΔC^_T_ method. We used the same gene expression assays for *PER1*-*3* and *BMAL1* and the control genes *B2M* and *PPIA* as previously described^21^. In addition, we studied the expression of *REV-ERBα* (Assay ID: Hs00253876_m1).

For each sample from a given subject, the expression of each clock gene was quantified relative to a calibrator RNA sample made up of a mix of RNA from all the samples obtained at baseline and following night shifts for this given subject. Data were transformed into Z-score for each subject (Z = (x − μ)/σ where x is the raw value, μ is the combined mean of both baseline and night shift conditions and σ is the combined standard deviation of both baseline and night shift conditions). This normalization process allowed comparing the amplitudes of gene expression rhythms between baseline and night shift conditions.

### Statistical analyses

All data are presented as mean ± SEM, except demographic data (age, body mass index, chronotype) reported as mean ± SD. Values of *P* ≤ 0.05 were considered significant. Data regarding participants (age, BMI, chronotype score, sleep duration, and light intensity) were analyzed with parametric or non-parametric t-tests.

Since the experimental protocol was based on participants’ habitual sleep times, circadian rhythms were analysed relative to each participant’s wake time at baseline. This allowed assessing circadian rhythms and their changes during the study relative to their baseline position (Figs 2, 3 and 4). For illustrative and analysis purposes, bedtimes and wake times were assigned a relative clock time of 0:00 and 8:00, respectively.

Harmonic regressions were applied to clock gene, cortisol, and melatonin data (nonlinear mixed-effect procedure using *nlmixed* SAS procedure, SAS Institute, Cary, NC, USA)^7,21,42^. A random factor was used to account for the inter-individual variability in the average cortisol and melatonin levels, but not for clock gene levels as they were z-score transformed. Cosine functions, including a mesor, amplitude, and phase, were used to model a single (24-h rhythm), dual (24-h + 12-h rhythms), or triple (24-h + 12-h + 6-h rhythms) harmonic regression. The *nlmixed* SAS procedure was performed on individual data (not averaged across subjects) and included all subjects independent on whether the individual data of a given subject displayed a significant rhythm or not. The results of the *nlmixed* SAS procedure were considered as the group results. In addition, we applied harmonic regressions on individual data (user-defined equation using Prism 6; GraphPad, La Jolla, CA, USA) and both individual and group results allowed us to statistically assess circadian rhythms at baseline and following night shifts. All statistical tests described thereafter used data obtained or derived from harmonic regressions applied to group data unless stated otherwise. Harmonic regressions applied on individual data were used for graphic representations in Figs S1 and S2 and for statistical assessment using a two sample t-test between percent of differences of percentages of rhythmic individuals for all genes between control and bright light groups under the day- and night-oriented schedules. To facilitate the comparison between and within different subjects, the “period” parameter of the regression we set at 24 h. Individual and group rhythms were significant when the 95% confidence interval of the fitted 24-h and/or 12-h amplitude did not include the zero value^21^ (confirmed by *P* values given by the *nlmixed* process for group rhythms).

A single-harmonic regression was used to assess rhythms of clock gene expression with the fitted phase corresponding to the time of maximal expression^21^. As previously shown, unimodal regressions are commonly used to analyze cortisol curves^15,21^. However, in this study, a visual inspection indicated that cortisol levels followed a bimodal pattern in the control group under a night-oriented schedule possibly resulting from the scheduling of sleep. Due to the bimodal nature of cortisol data and knowing that dual-harmonic regressions have been previously proposed to analyze cortisol data^43^, we decided to compare cortisol acrophases obtained by single-harmonic regression to those obtained from dual-harmonic regressions to decide which method was the most adapted to analyze cortisol group data. While the single-harmonic regression provided directly a fitted phase corresponding to the time of cortisol maximum, we had to use a two-step approach to obtain time of cortisol maximum using the dual-harmonic regression. This type of regression provided two fitted phases (main and secondary) and we used the main one to calculate the main composite acrophase (*via* a manual process) as previously published^7,21,40^. Using acrophases obtained with both methods, we calculated the group phase shifts (see below) between baseline and night shift conditions for both control and bright light groups and found they were similar (data not shown). Therefore, we used results obtained from dual-harmonic regressions to perform the statistical analyses described thereafter.

A 3-harmonic regression was used to assess rhythms of melatonin^7,21,44^ with the time of fitted phase corresponding to the time of maximal melatonin levels. Both 2- and 3-harmonic regressions provided fitted amplitudes and phases that were then used to calculate composite phases.

Phase shifts were calculated at the level of the group as the initial phase obtained at baseline (φ_1_) – final phase under the night shift condition (φ_2_). To statistically assess phase shifts with single-harmonic regression on group data, we used the F statistic provided by the *nlmixed* procedure taking into account the repeated measure design of the experiment. We used this type of analysis using group data rather than a more classical statistical test on individual data as our sample size would have been too low for some of the clock genes. Indeed, for several individuals, there was no significant rhythm either under the day-oriented schedule or the night-oriented schedule preventing us to include data from these individuals. All group rhythms were significant and were used for these analyses. For melatonin and cortisol, the phases obtained were composite phases of the group data. As described above, composite phases were not directly generated by the *nlmixed* procedure preventing us to use F statistic. Instead, we applied unpaired t-tests to analyze phase shifts.

The amplitude of each group rhythm was defined as the mean-to-trough difference of the 24-h harmonic of the regression^45^ and therefore, F-statistic was used to assess amplitude changes for clock gene expression, cortisol, and melatonin levels.

A cross-correlation analysis was performed to quantify the temporal relationship between cortisol levels and *PER1* expression rhythms observed in the control group under the night-oriented schedule.

## Electronic supplementary material

Supplementary Figures 1 and 2 and Supplementary Table 1
