# Supplementary material for: Rapid resetting of human peripheral clocks by phototherapy during simulated night shift work
Source: Sci Rep. 2017 Nov 24;7:16310. doi: 10.1038/s41598-017-16429-8 (PMC5701225; doi:10.1038/s41598-017-16429-8)
Supplement: Supplementary file 1 — Supplementary Figures 1 and 2 and Supplementary Table 1 [file 41598_2017_16429_MOESM1_ESM.pdf]

Rapid resetting of human peripheral clocks by phototherapy during simulated night shift work

Marc Cuesta, Philippe Boudreau,

Nicolas Cermakian & Diane B.

Boivin

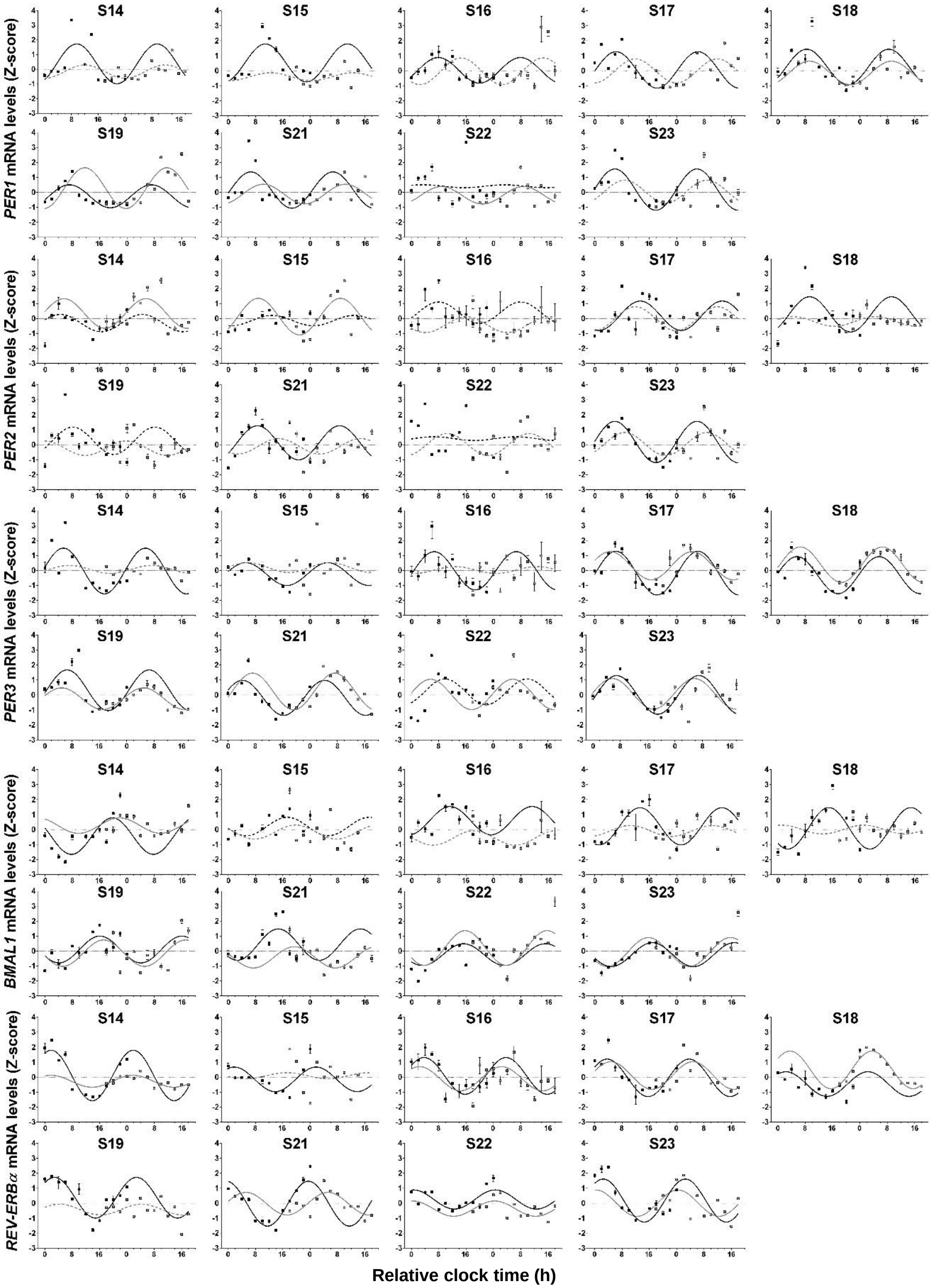

Figure S1. Individual circadian rhythms of clock gene mRNA expression in PBMCs in baseline and night shift in the control group. Mean levels (±upper and lower levels) and individual harmonic regressions of *PER1*, *PER2*, *PER3*, *BMAL1* and *REV-ERBα* are given as Z-scores in baseline (black) and following night shift (grey). Bedtimes and wake times were assigned a relative clock time of 0:00 and 8:00, respectively. For visualization purposes, the x-axis represents two 24-h cycles, and harmonic regressions were double plotted (over 48 h). Harmonic regressions are represented by solid lines when statistically significant and by dashed lines when non-significant.

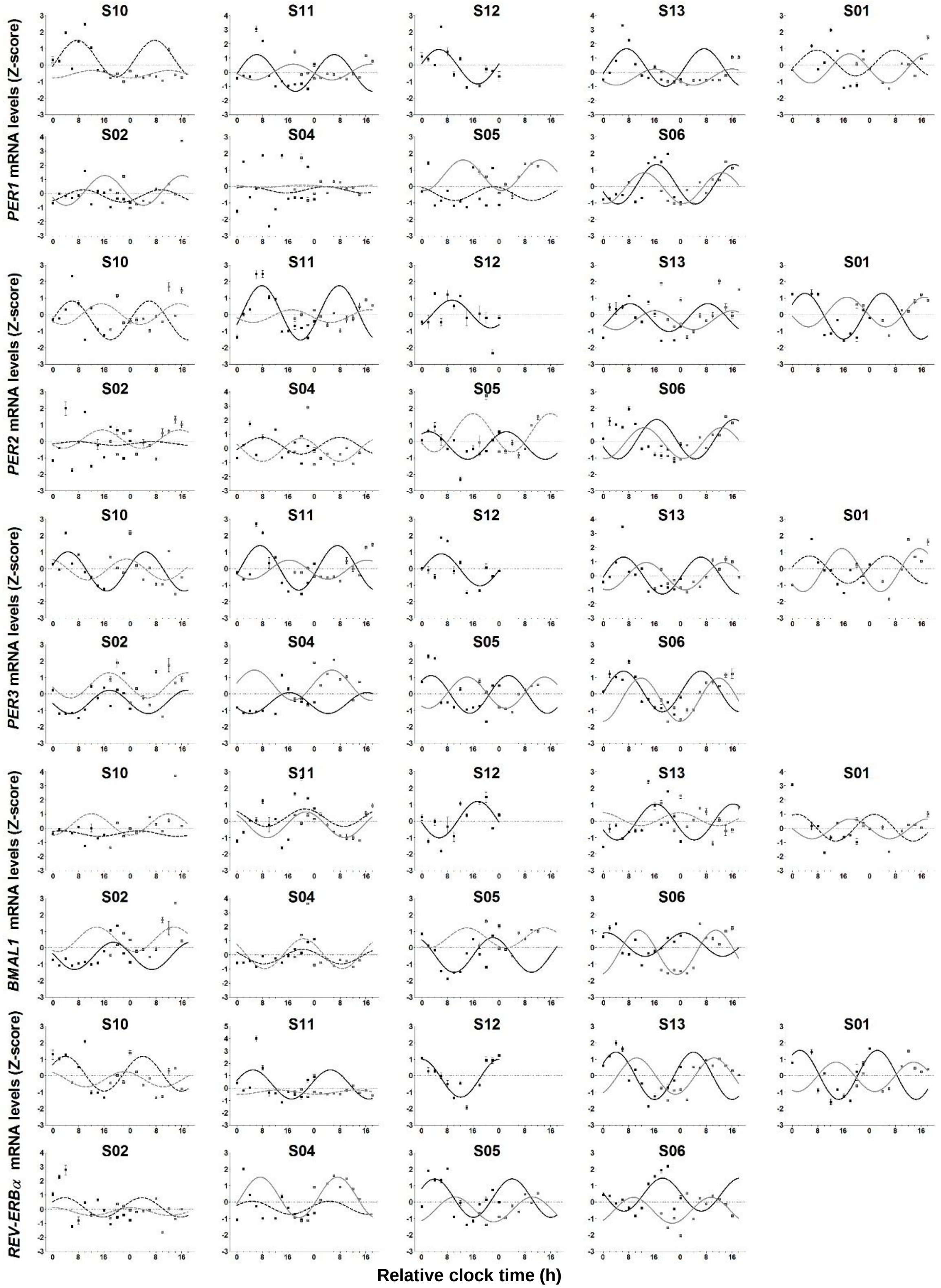

Figure S2. Individual circadian rhythms of clock genes mRNA expression in PBMCs in baseline and night shift in the bright light group. Mean levels (±upper and lower levels) and individual harmonic regressions of *PER1*, *PER2*, *PER3*, *BMAL1* and *REV-ERBα* are given as Z-scores in baseline (black) and following night shift (grey). Bedtimes and wake times were assigned a relative clock time of 0:00 and 8:00, respectively. For visualization purposes, the x-axis represents two 24-h cycles, and harmonic regressions were double plotted (over 48 h). Harmonic regressions are represented by solid lines when statistically significant and by dashed lines when non-significant.

Table S1. Demographic data

Subject	Group	Age	BMI	Time in bed	Time out of bed	Chronotype Type	Experimental period
S14	CTRL	21	20.4	23:57	7:59	Intermediate	August 2-7, 2012
S15	CTRL	20	19.6	22:31	6:31	Intermediate	May 15-20, 2013
S16	CTRL	27	20.7	0:30	8:16	Intermediate	May 27-June 1, 2013
S17	CTRL	18	20.4	23:00	7:02	Intermediate	June 11-17, 2013
S18	CTRL	29	23.0	0:01	8:01	Intermediate	June 26-July 1, 2013
S19 (woman)	CTRL	23	23.0	0:31	8:31	Intermediate	July 12-17, 2013
S20	CTRL	19	22.3	23:01	7:09	Moderately Morning	July 29-August 3, 2013
S21	CTRL	23	22.4	0:00	8:07	Intermediate	July 1-6, 2014
S22	CTRL	22	20.9	23:40	7:47	Intermediate	July 17-22, 2014
S23	CTRL	24	21.5	23:29	7:30	Intermediate	July 30-August 4, 2014
S01	BL	29	22.0	23:25	7:30	Moderately Morning	June 4-9, 2005
S02	BL	18	19.9	22:01	6:04	Intermediate	July 18-23, 2005
S04 (woman)	BL	22	21.2	0:00	8:01	Moderately Morning	July 25-30, 2005
S05	BL	23	23.6	23:25	7:28	Intermediate	August 3-8, 2005
S06	BL	30	23.5	23:58	8:07	Intermediate	August 11-16, 2005
S10	BL	30	21.5	0:00	8:01	Intermediate	June 3-8, 2012
S11	BL	28	23.5	22:26	6:30	Moderately Morning	June 17-22, 2012
S12	BL	19	22.5	23:00	7:04	Moderately Morning	July 2-7, 2012
S13	BL	22	24.5	23:01	7:02	Intermediate	July 8-13, 2012

CTRL: Control group; BL: Bright light group
